# Low admission serum albumin as prognostic determinant of 30-day case fatality and adverse functional outcome following acute ischemic stroke

**DOI:** 10.11604/pamj.2013.14.53.1941

**Published:** 2013-02-07

**Authors:** Sani Abubakar, Anas Sabir, Mohammed Ndakotsu, Maryam Imam, Mohmmed Tasiu

**Affiliations:** 1 Department of Medicine, Ahmadu Bello University Teaching Hospital, Zaria, Nigeria; 2 Department of Medicine, Usmanu Danfodiyo University Teaching Hospital, Sokoto, Nigeria; 3 Department of Pathology Usmanu Danfodiyo University Teaching Hospital, Sokoto, Nigeria; 4 Department of Physiology Usmanu Danfodiyo University Sokoto. Nigeria

**Keywords:** Stroke, outcome, serum albumin

## Abstract

**Introduction:**

Over 80% of stroke deaths occur in low-income and middle-income regions of the world. Identification of predictors of mortality is vital so that prompt therapeutic measures could be instituted to improve outcome. Previous studies have identified factors such as stroke severity, stroke type, older age, impairment of consciousness and hyperglycaemia as predictors of mortality for acute stroke but mortality remain high among patients hospitalized for acute stroke. The study objective was to determine the association between admission serum albumin levels and short-term outcome following acute ischaemic stroke in Nigerians.

**Methods:**

Consecutive first-ever acute ischaemic stroke patients were prospectively enrolled between February 2009 and May 2010. Stroke severity at presentation was determined using National Institute of Heath Stroke Score (NIHSS). Admission serum chemistry including albumin, were measured. Patients were then followed up for 30 days and outcome measures applied at the end of the study were 30-day mortality and functional outcome using the Modified Rankin Scale (MRS) and graded as favourable(MRS 0-3) or unfavourable(MRS 4-6). Relationship between serum albumin and stroke outcome was determined.

**Results:**

75 acute stroke cases were studied. Mean age was 57.68 ± 12.4 years. Outcome was favourable in 48% while 30-day case fatality was 17.3%. The mean age (61.13 years) of those with poor outcome was significantly higher than those with favourable outcome. Mean serum albumin (3.03g/dL) of those with favourable outcome was also significantly higher than (2.08g/dL) of those with unfavourable outcome (p=0.0001). Patients that died had significantly lower serum albumin (1.66g/dl) than survivors (p=0.0001).Receiver operating characteristics curve for optimal cut off point of serum albumin to predict survival or death within 30 days revealed area under the cure (AUC) of 0.870, p-value 0.0001, 95% C/I=0.759-0.982. Serum albumin of 1.55g /dL has sensitivity of 100% and specificity of 61.5%. NIHSS and serum albumin were predictors of poor outcome using multiple regression.

**Conclusion:**

Low admission serum albumin was an independent determinant of poor outcome.

## Introduction

It has been projected that by the year 2020, stroke will be the second leading cause of death and disability in developed regions of the world [1]. Globally about 15 million new stroke events occur every year, two-third of which occur in people living in low income and middle income countries. Demographic transition resulting from adaptation of westernized lifestyle is also likely to increase the burden of stroke in developing economies [1]. In general; early in-hospital mortality from stroke is usually directly related to the stroke itself, whereas factors related to hospitalization and complication of being hospitalized influence death later in the course of acute stroke. Previous studies have identified factors such as stroke severity, stroke type, older age, impairment of consciousness and hyperglycaemia as predictors of mortality following acute stroke [2–5]. but the mortality from stroke remain high among patients hospitalized for acute stroke in Nigeria, as a third of patients are dead by the forth week while a third remain permanently disabled [6]. Serum albumin is a multifunctional protein which along with other properties also offers neuroprotective effects. Experimental animal studies have shown human albumin in moderate to high doses to be a promising neuroprotectant in focal and global cerebral ischaemia and traumatic brain injury [7–9]. To the best of our knowledge, the effect of serum albumin on stroke outcome in Nigerians is not known. The study objective was to determine the association between admission serum albumin levels and short-term outcome following acute ischaemic stroke in Nigerians.

## Methods

The study was carried out at Usmanu Danfodioyo University Teaching Hospital (UDUTH) Sokoto, in which consecutively presenting first-ever acute ischemic stroke cases were prospectively enrolled. UDUTH is a tertiary heath care institution situated in Sokoto, a predominately urban setting in north western Nigeria with a population of about 427760 (2006 census). It is a 585 in-hospital bed capacity with 96 of these been in the medical wards. First-ever acute stroke cases that fulfilled the predetermined inclusion criteria; all prospective patients admitted to UDUTH within 72hours of onset of stroke symptoms, Patients with CT-Scan confirmed acute stroke not in keeping with haemorrhagic stroke or subarachnoid haemorrhage. Those with appropriate clinical scenario with normal brain CT-Scan result were also considered as ischemic stroke and were enrolled after informed consent. Patients with evidence of renal or liver disease as well as those with fever or infections were excluded.

Those patients that signed against medical advice after enrolment were also excluded.

The patients were actively recruited by the investigators and had a thorough clinical evaluation as contained in the structured questionnaire designed for the study. Stroke severity on admission was determined using the National Institute of Health Stroke Score (NIHSS) [10] Brain CT-Scan was routinely requested in all instances as part of stroke management protocol of the unit, to confirm the diagnosis of stroke. Electrocardiography and echocardiography were done for patients with suspected cardio-embolic stroke or underlying heart disease. Other investigations such as serum albumin, Total white cell counts (WBC), and serum creatinine as well as random blood sugar were all done at presentation. Blood samples were collected immediately after admission before administration of any medication or intravenous fluid and subsequently sent to the laboratory for prompt evaluation. Serum albumin was measured using Abbott Aeroset Bromocresol Green (BCG).

All patients had standard stroke management in accordance with guidelines of the neurology unit of UDUTH, in conformity with international standards. None of the patients had thrombolytic therapy. The patients were evaluated routinely for assessment of progress and development of complications. The complications noted were promptly treated. The study did not in any way interfere with the treatment of the patients. Patients were then followed up for maximum of 30 days.

Outcome measures applied at the end of the 30 days were, functional status using modified Rankin scale (MRS) [11] and 30-day mortality. Patients discharged from the hospital before this period were asked to visit out-patient department at weekly interval. Telephone inquiry was used to confirm outcome in patients who failed to turn up for the outpatient visit.

Hypertension was taken as positive history of hypertension, use of anti-hypertensive drugs or persistently elevated blood pressure (>140/90 mmHg) while on admission. Diabetes mellitus was regarded as a positive history, use of hypoglycemic agents or a fasting plasma glucose of >7.0mmol/L on two occasions. Stroke was defined as a clinical syndrome of sudden onset of rapidly developing symptoms and signs of focal or global cerebral deficit with symptom lasting more than 24 hours or leading to death with no apparent cause other than vascular origin [12]. Modified Rankin Scale was used to assess outcome at the end of the study and was graded from 0 to 6.0=recovered with no symptoms while 6 corresponds to death. Patients with score of 0-3 were thus categorized as favourable (good) outcome while 4-6 as unfavourable (poor) outcome [13]. Formal education means duration of formal education in years (primary=6 secondary=12 and tertiary= ≥13).

Data obtained was analysed using SPSS 18.0, independent samples T-test was used to compare means while Chi-square test for comparing frequencies and percentages. Multiple (binary) logistic regressions were used to determine relationship of serum albumin with stroke outcome in the presence of other covariates (age, WBC, NIHSS,RBS and duration of formal education). Receiver Operation curve (ROC) was used to determine sensitivity and specificity of albumin as determinants of 30-day mortality. P value less than or equal to 0.05 were considered statistically significant.

## Results

A total of 129 consecutively presenting acute stroke cases were prospectively recruited of which 75 fulfilled the predetermined inclusion criteria. 54 were excluded from the study for several reasons; 29 acute stroke cases presented after 72 hours of onset of stroke symptoms, 5 patients had clinical evidence of liver disease at presentation while 2 patients were on treatment for arthritis prior to stroke onset. 14 patients had previous history of stroke and 4 patients with inadequate follow up were all excluded from the study. A total of 75 stroke cases were thus included in the study comprising 39(52%) males and 36(48%) females with male: female ratio of 1.08:1. The age of the patients ranged from 20-87years with a mean of 57.68±12.4years. Systemic hypertension was the most common modifiable risk factor for stroke followed by diabetes as shown (Table 1).

**Table 1:** Baseline characteristics of the patients stratified by sex

Characteristics	Total (N=75)	Female (N=36)	Male (N=39)	P-value
**Gender**	75	36	39	
**Age in years: mean (±SD)**	57.68(12.4)	60.91 (10.5)	54.69(13.3)	0.029
**Age range(yrs)**	20-87	40-80	20-87	
**Adm BP mean (±SD) mmHg**				
SBP	159.3(34.38)	162.8(32.3)	156.15(36.3)	0.4
DBP	92.53(19.1)	91.1(18.94)	93.8(19.4)	0.54
**Risk factor profile; N (%)**				
Hypertension	56(74.7)	31(86.1)	25(64.1)	0.40
Diabetes	8(10.7)	6(16.7)	4(10.3)	0.48
Cardiac diseases	5(6.7)	3(8.3)	2(5.1)	0.60
Cigarette	5(6.7)	1(2.8)	4(2.6)	0.22
Alcohol	1(1.3)	0(0)	1(2.6)	0.34
**Adm NIHSS:mean (±SD)**	9.6(3.9)	9.7(3.2)	9.54(4.39)	0.84

SBP=systolic blood pressure, DBP=diastolic blood pressure, SD= standard deviation, N=number, adm=admission,

The overall outcome in terms of morbidity was favourable (good) in 48% ( N=36). The mean age (61.13 ±10.3 years) of those with poor outcome was significantly higher than (53.94±13.4 years) those with favourable (good) (Table 2).

**Table 2:** Baseline characteristics stratified by 30-day morbidity (MRS category)

Characteristic	Total (N=75)	Poor outcome (N=39)	Good outcome (N=36)	P-value
Age in years: Mean (±SD)	57.68(12.4)	61.13 (10.3)	53.94 (13.4)	0.01
Admiss NIHSS: Mean (±SD)	9.6 (3.9)	11.18 (3.8)	7.9 (3.2)	0.0002
Albumin g/dL: Mean (±SD)	2.54 (0.79)	2.08 (0.67)	3.03 (0.61)	<0.0001
Creatinine mg/dL: Mean (±SD)	0.98 (04)	0.9 (0.4)	1.0 (0.4)	0.24
WBC X 10^9^ /L: Mean (±SD)	8.3 (3.2)	9.8 (2.4)	6.6 (3.1)	<0.0001
RBS mmol/L: Mean (±SD)	6.4(3.2)	6.7 (3.8)	6.2 (2.3)	0.50
SBP mmHg: Mean (±SD)	159.3(34.4)	160.2 (30.9)	158.1 (38.2)	0.8
DBP mmHg: Mean (±SD)	92.5 (19.1)	93.8 (18.9)	91.1 (19.5)	0.54

SD =standard deviation, SBP=systolic blood pressure, WBC= total white cell count, DBP=diastolic blood pressure, NIHSS= national institute of Health stroke score, RBS=random blood sugar, admiss=admission

Thirteen (13) patients died during the 30 days follow-up thus making the 30-day case fatality to be 17.3%. The mean admission random blood sugar was significantly lower in those that survived (Table 3).

**Table 3:** Clinical and biochemical variables and relationship with 30 days mortality

Characteristics	Total (N=75)	Alive (N=62)	Death (N=13)	P- value
Age in years: mean (±SD)	57.68(12.4)	57.2(12.7)	60.0(10.0)	0.5
Adm NIHSS: Mean (±SD)	9.6(3.9)	8.8(2.9)	13.6(5.2)	<0.0001
Albumin g/dL: Mean (±SD)	2.54(0.79)	2.72(0.68)	1.66(0.71)	<0.0001
Creatinine mg/dL: Mean (±SD)	0.98(0.4)	1.0(0.4)	0.9(0.2)	0.3
WBC X 10^9^ /L: Mean (±SD)	8.3(3.2)	7.9(3.1)	10.3(2.9)	0.01
Adm RBS mmol/L: Mean (±SD)	6.4(3.2)	6.0(2.3)	8.4(5.3)	0.01
Adm SBP mmHg: Mean (±SD)	159.3(34.4)	158.2(34.3)	164.6(35.7)	0.55
Adm DBP mmHg: Mean (±SD)	92.5(19.1)	92.1(19.2)	94.6(17.6)	0.7

SD =standard deviation, ESR=erythrocyte sedimentation rate, SBP=systolic blood pressure, WBC= total white cell count, DBP=diastolic blood pressure, NIHSS= national institute of Health stroke score

Serum albumin of stroke patients above 65 years was not significantly different from those below 65 years as shown in (Table 4).

**Table 4:** Univariate association between admission serum albumin and age with outcome

	<3.0g/dL	3.0-3.5g/dL	>3.5g/dL	p-value
**Age in years**				0.83
≤65	2	18	11	
>65	9	7	6	
**Outcome**				0.004
Alive	22	23	17	
Death	11	2	0	

**Figure 1:**
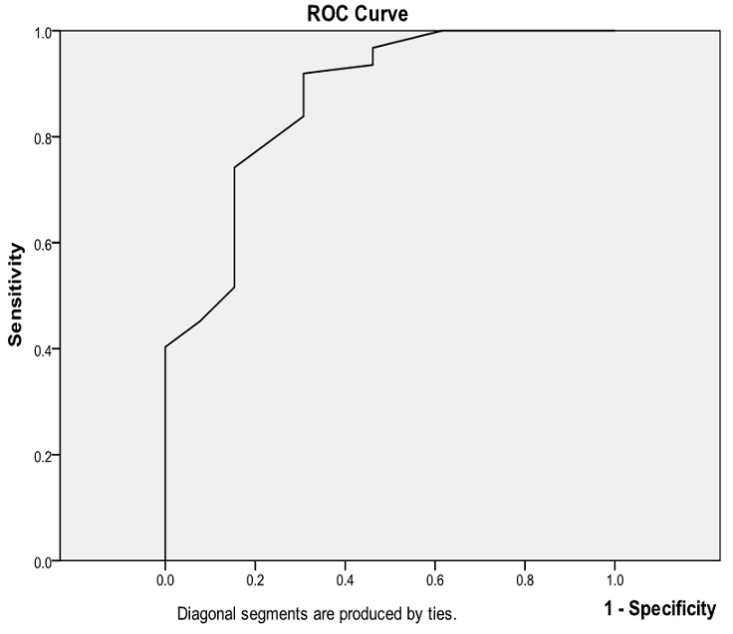
Receiver operating characteristic (ROC) curves to show optimal cut off point of serum albumin g/dL to predict whether patients will survive or die within 30-day of stroke onset

The analysis was also performed for predicting outcome (alive) corresponding to various level of serum albumin. The cut off points of ROC curves were 1.55g/dL (sensitivity 100%,specificity 61.5%), 2.35g/dL (sensitivity 93.5%, specificity 46.2%), 2.56g/dL (sensitivity 84% specificity 30.8%), while the area under the curve (AUC) is 0.87 (95% CI 0.759-0.982) as shown in Figure 1. In a multivariate logistic regression (Table 5) admission stroke severity measured by NIHSS and serum albumin correlated with 30 days mortality in the presence of other variables (age, WBC, NIHSS, RBS and duration of formal education).

**Table 5:** Determinants of 30-day mortality using multivariate logistic regression

Variable	S.E	95% CI	Correlation coefficient	p-value
Age ( years)	0.41	0.928-1.169	0.595	0.469
Serum albumin	-3.07	0.004- 0.496	-0.163	0.011
WBC	-0.26	0.466-1.265	-0.272	0.300
NIHSS	0.67	1.126-3.374	-0.138	0.017
RBS	0.18	0.872-1.647	-0.298	0.263
Formal education	-0.18	0.074-9.509	-0.071	0.887

WBC=white blood cell count, RBS=random blood sugar, NIHSS=national institute of health stroke score, formal education=duration of formal education

## Discussion

The patients were predominately middle-aged (mean 57years) that is a decade lower than peak age of stroke in industrialized countries [14]. However this is similar to finding in previous studies in the region [15, 16] and also comparable to finding two decades ago in south western part of Nigeria [17]. This study showed that females were significantly older than the males and presented with more severe stroke, a finding that is in agreement with those of Gall SL et al [18]. Females presenting with more severe stroke suggest that women may have lower accessibility to hospital than men for cultural and religious reasons; alternatively stroke may be more severe in females than males for unexplained reasons. Hypertension and diabetes mellitus were the most common modifiable risk factor for stroke, a finding that we have described earlier in a retrospective study at the same study site [15]. More than half (52%) of the patients had poor outcome (measured by MRS ≥ 4), at the end of the 30 days follow up. Factors that were noted to have influenced outcome were, patients age at time of stroke, admission stroke severity, serum albumin and total white cell count. Increasing age was associated with worse functional outcome and 30 days case fatality. This further confirm findings in previous studies where increasing age was associated with more severe stroke at presentation, increased duration of hospital stay and 28 days mortality [19].

Albumin is a non-glycosylated plasma protein synthesized primarily in the liver. It is a protein involved in the transport of small molecules in the blood and plays a key role in restricting fluid leakage from the vasculature into the tissue [20]. Elevated level of serum albumin is related to haemoconcentration and reduced level is associated with malnutrition, chronic inflammatory diseases representing a negative acute phase protein [21]. This study showed that low serum albumin (at presentation) is associated with worse outcome and the mean serum albumin of those that died was significantly lower compared to the survivors. This conforms to the finding of Dziedzic T et [22] al in which a relatively high serum albumin level in acute stroke patients decreased the risk of poor outcome [22]. Various mechanisms have been proposed to explain the effect of serum albumin on stroke outcome. It is suggested that albumin decreases the hematocrit levels, impedes erythrocyte aggregation and reduces the erythrocyte sedimentation [23]. Albumin also antagonize thrombosis, stagnation and leukocyte adhesion within the post capillary microcirculation in early reperfusion phase of stroke thus offering neuro protection in stroke patients [24].

Serum albumin has been associated with adverse vascular events in patients with cardiac [25] and renal diseases [9]. Serum albumin has neuroprotective effect that is mediated by its multitude of actions including anti-oxidant properties, modulation of endothelial functions and venular perfusion [9, 25]. Albumin ameliorates brain swelling, enhancing blood flow to sub-occlusive microvascular leision, maintaining vascular patency and preventing re-occlusion after successful thrombolysis [26].

Serum albumin is regulated by factors influencing protein synthesis, breakdown, leakage to the extravascular space and food intake. In clinical practice serum albumin is often considered a marker of nutritional status and a negative phase protein that decrease in concentration during injury and sepsis [27]. Serum albumin is a negative acute phase protein and concentration falls between day 1 and day 7 after stroke onset [28], which could possibly explain the finding of marginally low serum albumin in our series. Alvarez-perez FJ et a l [29] looked at potential association between lower albumin level and cardio-embolic stroke and found that mean serum albumin of patients with acute ischaemic stroke was significantly lower than controls, and stroke patients that died or remained dependent at discharge (from hospital) was significantly lower than survivors. This is similar to finding of relatively low serum albumin in our stroke patient. There was no significant association between age and admission serum albumin in this study population as opposed to finding by *TT Idicula* [30] where elderly stroke patients had significantly lower admission serum albumin. This could possibly be explained by the fact that our cohort was a relatively younger population.

We acknowledge the modest size of the sample studied and indicate that our findings, though preliminary in nature, provide a thrust for larger, more elaborate studies. However, we have illustrated that reduced serum albumin is an accompaniment of ischaemic stroke in our environment, correlates with stroke severity and independently predicts adverse outcome including fatality and functional impairment.

## Conclusion

Low admission serum albumin and admission stroke severity measured by NIHSS were an independent determinant of poor outcome.
